# Indole derivatives display antimicrobial and antibiofilm effects against extensively drug-resistant *Acinetobacter baumannii*

**DOI:** 10.1128/spectrum.03388-24

**Published:** 2025-04-15

**Authors:** Junwei Li, Lulin Xie, Fei Lin, Baodong Ling

**Affiliations:** 1 Key Laboratory of Structure-Specific Small Molecule Drugs at Chengdu Medical College of Sichuan Province, School of Pharmacy, Chengdu Medical College 74787, Chengdu, China; 2 Department of Pharmacy, Clinical Medical College and The First Affiliated Hospital of Chengdu Medical College 580504, Chengdu, China; The University of North Carolina at Chapel Hill, Chapel Hill, North Carolina, USA

**Keywords:** *Acinetobacter baumannii*, extensive drug resistance, biofilm, indoles

## Abstract

*Acinetobacter baumannii* is a critical priority gram-negative bacterial species featured with multidrug resistance and biofilm formation. This study screened 46 indole derivative agents for their antimicrobial activities against clinical isolates of extensively drug-resistant *A. baumannii* (XDRAB) with various degrees of biofilm production. Three selected indole agents—5-iodoindole, 3-methylindole, and 7-hydroxyindole—were revealed to display potent antimicrobial and antibiofilm activity, including synergistic interplay with anti-*A*. *baumannii* antimicrobial drugs against XDRAB. Sub-inhibitory concentrations of these agents (particularly 7-hydroxyindole at 1/64 of MIC) not only inhibited XDRAB biofilm formation but also eradicated the mature biofilm. The survival rate of XDRAB-infected *Galleria mellonella* was improved with the treatment of 7-hydroxyindole. Mechanistically, 7-hydroxyindole was found to reduce the expression of quorum sensing/biofilm-implicated genes *abaI* and *abaR*. Together, the findings highlight the potential of indole derivatives against *A. baumannii* infections.

**IMPORTANCE:**

Extensively drug-resistant *Acinetobacter baumannii* (XDRAB) isolates pose a major public health threat to antimicrobial therapy and are highly prevalent in hospital settings. This study identified and characterized indole derivative agents for their antimicrobial and antibiofilm activities against XDRAB. Sub-inhibitory indole agents such as 7-hydroxyindole can both inhibit XDRAB biofilm formation and eradicate the mature biofilm. Indole agents warrant further investigation against hard-to-treat antimicrobial-resistant pathogens.

## INTRODUCTION

Infections associated with *Acinetobacter baumannii* pose a major threat to public health around the globe (1–4). This pathogen is featured with sophisticated mechanisms of antimicrobial resistance, which leads to significant intrinsic resistance and a propensity to develop acquired multidrug resistance (3, 5, 6). Indeed, extensively drug-resistant *A. baumannii* (XDRAB) is particularly worrisome for their contribution to hospital-acquired infections and impact on antibiotic therapy (4, 7, 8). The World Health Organization lists *A. baumannii* as one of the top critical priority pathogens that are of public health importance and require research, development, and strategies to prevent and control antimicrobial resistance (9). Various virulence factors and physiological traits are known to facilitate *A. baumannii* infection and adaptation to the hospital environment (10). A key risk factor from *A. baumannii*, including XDRAB, is the formation of bacterial biofilms (5, 10). The latter is reported to be associated with 65% of bacterial infections (11). Biofilm cells also produce much higher levels of resistance than their planktonic counterparts (12, 13). Furthermore, biofilms facilitate bacterial adaptation to hostile internal environments and enable evasion of the host immune system, resulting in recurrent infections and significantly complicating clinical therapy (14, 15). Thus, intervention strategies against biofilms are of important potential in combating infections (13, 16) . Our previous study showed that antimicrobial drugs (azithromycin and rifampicin) and other agents (zinc lactate, stannous fluoride, and furanone) were active *in vitro* at preventing biofilm formation of XDRAB but exhibited minimal activity against mature biofilms (17). Earlier research on antibiofilm agents has predominantly focused on inhibiting biofilm formation, with limited reports on agents capable of effectively disrupting or eradicating mature biofilms (18, 19).

As heterocyclic organic agents, indoles are widespread in nature and function as important signaling molecules in diverse prokaryotes and eukaryotes (20, 21). They also possess medicinal potential for therapeutic interventions to diverse medical conditions, including microbial infections (22, 23). In recent years, attention has been drawn to indoles for their diverse and potent pharmacological properties against microbes (24–27). Studies have revealed that indole derivatives possess antimicrobial and biofilm-inhibiting activities against various pathogenic microorganisms, including *Escherichia coli* (28, 29), *Pseudomonas aeruginosa* (29), *Vibrio cholerae* (30), and *Candida albicans* (31, 32). Several *bis*-indole agents were demonstrated to exhibit activities against multidrug-resistant gram-positive and gram-negative bacterial species including *A. baumannii* (33, 34). However, the potential of indoles against *A. baumannii* biofilms and related mechanisms remains largely unknown.

In this study, we screened and identified indole derivative agents with respect to their antimicrobial and antibiofilm activities against clinical isolates of XDRAB. The findings revealed that several indole agents not only exhibited significant anti-XDRAB activity but also were able to reduce or eradicate XDRAB biofilms, highlighting the potential of indole derivatives in controlling *A. baumannii* infections.

## RESULTS

### Anti-XDRAB activity of antimicrobial drugs and indole derivatives

A total of 70 clinical isolates were examined for their antimicrobial susceptibility phenotypes, followed by obtaining 81% (57/70) of isolates belonging to XDRAB (Table 1 and Tables S1 and S2) (35). Antimicrobial minimal inhibitory concentration (MIC) values are included in Table 1 and Table S2. The rates of resistance to individual antimicrobial agents ranged from 34% to 86% (except no resistance to tigecycline when using the resistant breakpoint of MIC ≥ 8 µg/mL for *Enterobacterales* [36]) (Table S3). These clinical isolates were also assessed for their biofilm formation abilities by grouping them as strong (17% [12/70]), medium (14% [10/79]), weak (60% [42/70]) and no (9% [6/70]) biofilm producers (Table S4).

**TABLE 1:** Antimicrobial activities of 16 antimicrobial drugs and three indole agents against XDRAB

Agents	MIC (μg/mL) for XDRAB					
	A19	A35	A43	A46	A49	A50
Antimicrobial drugs						
Imipenem	16	64	128	128	16	128
Meropenem	64	64	32	64	16	16
Ampicillin	>1,024	>1,024	>1,024	>1,024	>1,024	>1,024
Ampicillin-sulbactam (2:1)	256	128	256	256	32	64
Ceftazidime	128	64	64	128	1,024	128
Cefotaxime	256	256	512	512	1,024	512
Cefoperazone-sulbactam (2:1)	256	256	256	256	256	256
Amikacin	>1,024	>1,024	>1,024	>1,024	>1,024	>1,024
Gentamicin	>1,024	>1,024	>1,024	>1,024	>1,024	>1,024
Ciprofloxacin	64	128	16	32	32	64
Levofloxacin	16	8	16	16	8	16
Doxycycline	64	32	64	64	32	64
Minocycline	16	16	8	8	4	8
Tetracycline	512	512	512	512	512	512
Tigecycline	2	2	2	2	1	2
Polymyxin B	4	8	4	1	4	4
Indoles						
5-Iodoindole	64	64	64	64	64	64
3-Methylindole	64	64	64	64	64	64
7-Hydroxyindole	512	512	512	512	512	512

**TABLE 2:** Synergistic antimicrobial effect of indole agents and antimicrobial drugs on six XDRAB strains

Indoles	Antimicrobials	FICI^a^	Interaction^b^ (%)		
			Synergy	Additivity	Indifference
5-Iodoindole	Meropenem	0.375–1	50	50	0
	Imipenem	0.25–0.75	50	50	0
	Cefoperazone-sulbactam (2:1)	0.375–1	33	67	0
	Ampicillin-sulbactam (2:1)	0.125–0.625	83	17	0
	Tigecycline	0.625–1.25	0	67	33
	Polymyxin B	0.625–1.25	0	67	33
3-Methylindole	Meropenem	0.1875–0.625	83	17	0
	Imipenem	0.1875–0.625	83	17	0
	Cefoperazone-sulbactam (2:1)	0.25–0.625	50	50	0
	Ampicillin-sulbactam (2:1)	0.375–0.625	50	50	0
	Tigecycline	0.625–1.5	0	17	83
	Polymyxin B	0.25	100	0	0
7-Hydroxyindole	Meropenem	0.188–0.562	83	17	0
	Imipenem	0.156–0.312	100	0	0
	Cefoperazone-sulbactam (2:1)	0.094–0.312	100	0	0
	Ampicillin-sulbactam (2:1)	0.047–0.625	83	17	0
	Tigecycline	0.625–1.5	0	17	83
	Polymyxin B	1.25	0	0	100

^a^ FICI, fractional inhibitory concentration index.

^b^ No antagonism observed.

**Fig 1:**
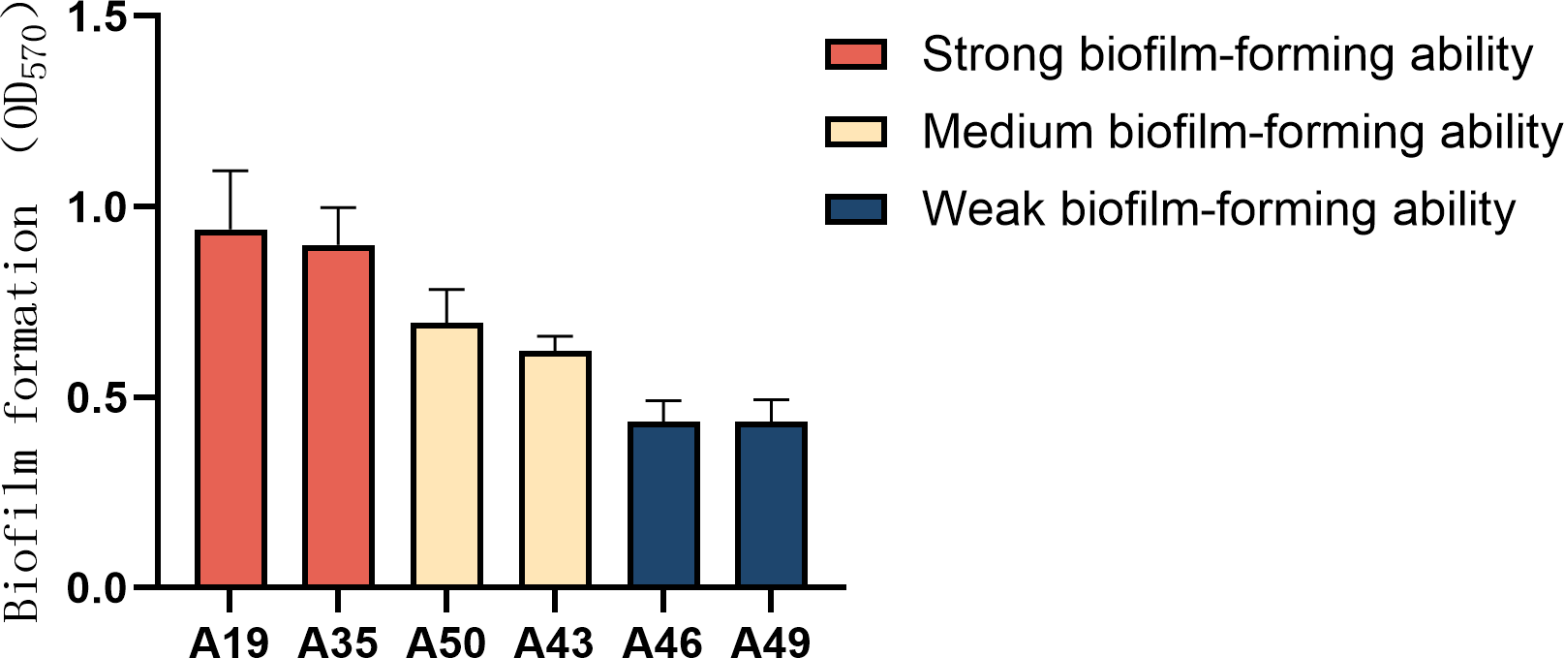
The biofilm formation abilities of six XDRAB isolates (*n* = 6, $\overset{-}{x}$ ± *s*).

Based on biofilm formation abilities, six XDRAB strains (i.e., A19, A35, A43, A46, A49, and A50; two isolates from each of the strong, medium, and weak biofilm producers) were selected for determining antimicrobial activities of indole derivative agents. The resistance profiles and biofilm formation of these six isolates are shown in Table 1 and Fig. 1, respectively. Notably, these isolates exhibited resistance to antipseudomonal carbapenems, extended-spectrum cephalosporins, β-lactam-β-lactamase inhibitor combinations, aminoglycosides, antipseudomonal fluoroquinolones, tetracyclines (except tigecycline), and polymyxin B (except one isolate being susceptible with MIC of 1 µg/mL) (37) but were susceptible/intermediate to tigecycline.

**Fig 2:**
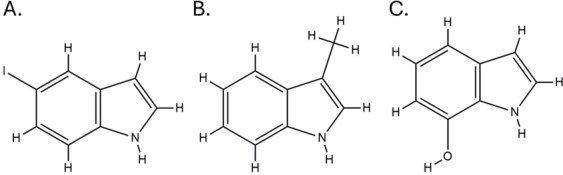
Structures of three indole agents—5-iodoindole (**A**), 3-methylindole (**B**), and 7-hydroxyindole (**C**)—that exhibit anti-XDRAB activity.

Subsequently, we screened a large number of indole agents (Table S2) for their activities against the aforementioned six XDRAB isolates (Table 1). Of the 46 indole derivatives tested (Table S1), 37 exhibited varying degrees of activity against XDRAB isolates, with MIC values ranging from 64 to 1,024 µg/mL (Table S5). Notably, MICs of 5-iodoindole, 5-fluoroindole, 6-bromoindole, and 3-methylindole were 64 µg/mL, while the MIC of 7-hydroxyindole was 512 µg/mL (Table 1 and Table S5). Three indole agents—5-iodoindole, 3-methylindole, and 7-hydroxyindole—were further investigated in this study, and their chemical structures are given in Fig. 2.

### Synergistic antimicrobial effects of indole agents with conventional antimicrobial drugs

To understand the clinical therapeutic potential of indoles, we tested the synergistic antimicrobial effect of indole derivatives in combination with drugs used in the clinical treatment of *A. baumannii* infection. The results showed that three indoles tested—5-iodoindole, 3-methylindole, and particularly 7-hydroxyindole—exhibited synergistic antimicrobial activities with carbapenems or β-lactam-β-lactamase inhibitor combinations against six XDRAB strains (Table 2). 3-methylindole and polymyxin B also showed synergy, while indoles and tigecycline produced no synergistic interaction. However, no antagonism among the tested combinations was observed (Table 2).

### Effects of indole agents on XDRAB biofilm

**Fig 3:**
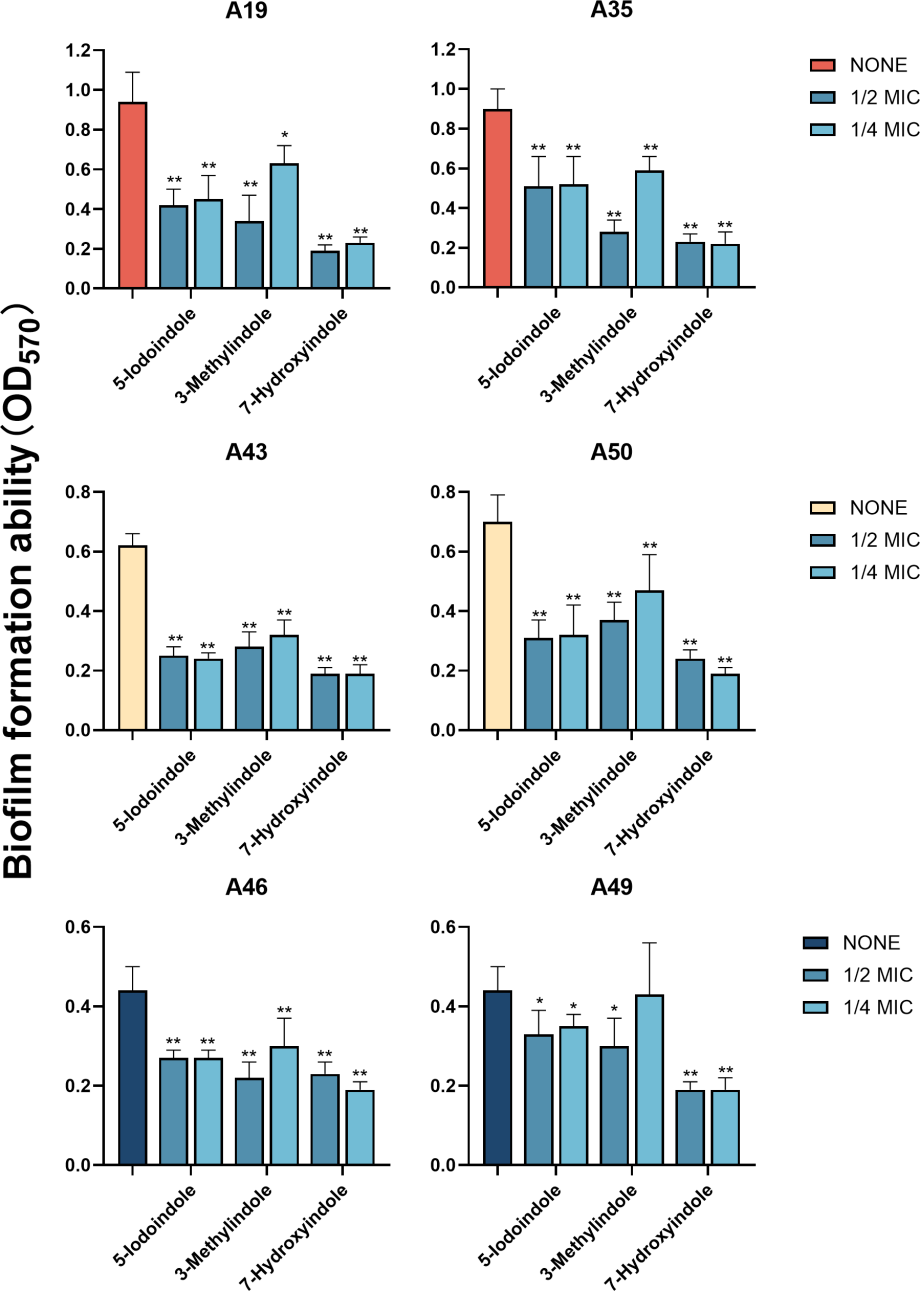
Biofilm formation inhibition of six XDRAB isolates (**A19, A35, A43, A50, A46, and A49**) by 5-iodoindole, 3-methylindole, and 7-hydroxyindole at sub-MIC levels (*n* = 6, $\overset{-}{x}$ ± *s*; *P < 0.05, **P < 0.01 versus none group).

**Fig 4:**
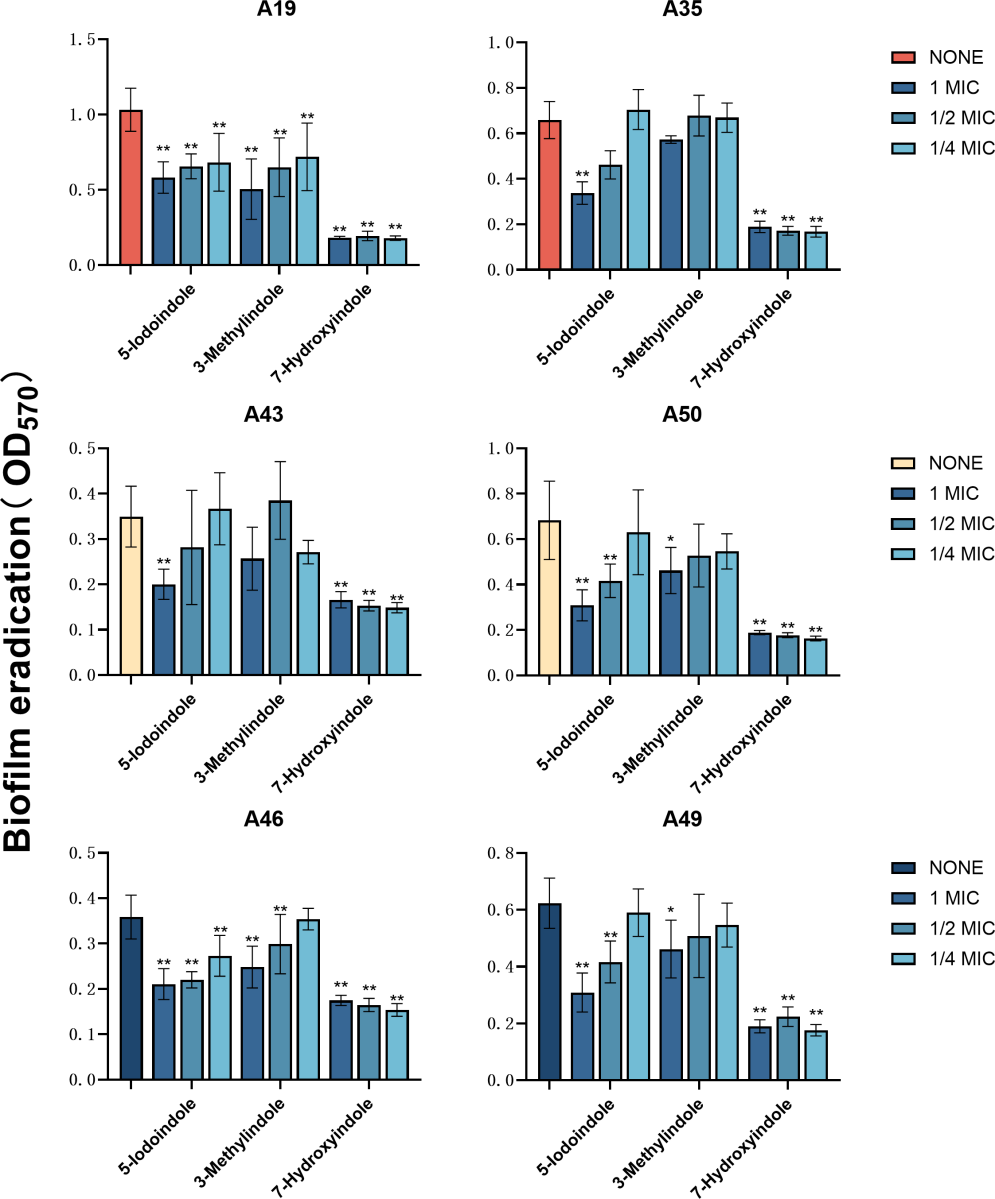
Biofilm eradication of six XDRAB isolates (**A19, A35, A43, A50, A46, and A49**) by 5-iodoindole, 3-methylindole, and 7-hydroxyindole at sub-MIC levels (*n* = 6, $\overset{-}{x}$ ± *s*; *P < 0.05, **P < 0.01 versus none group).

**Fig 5:**
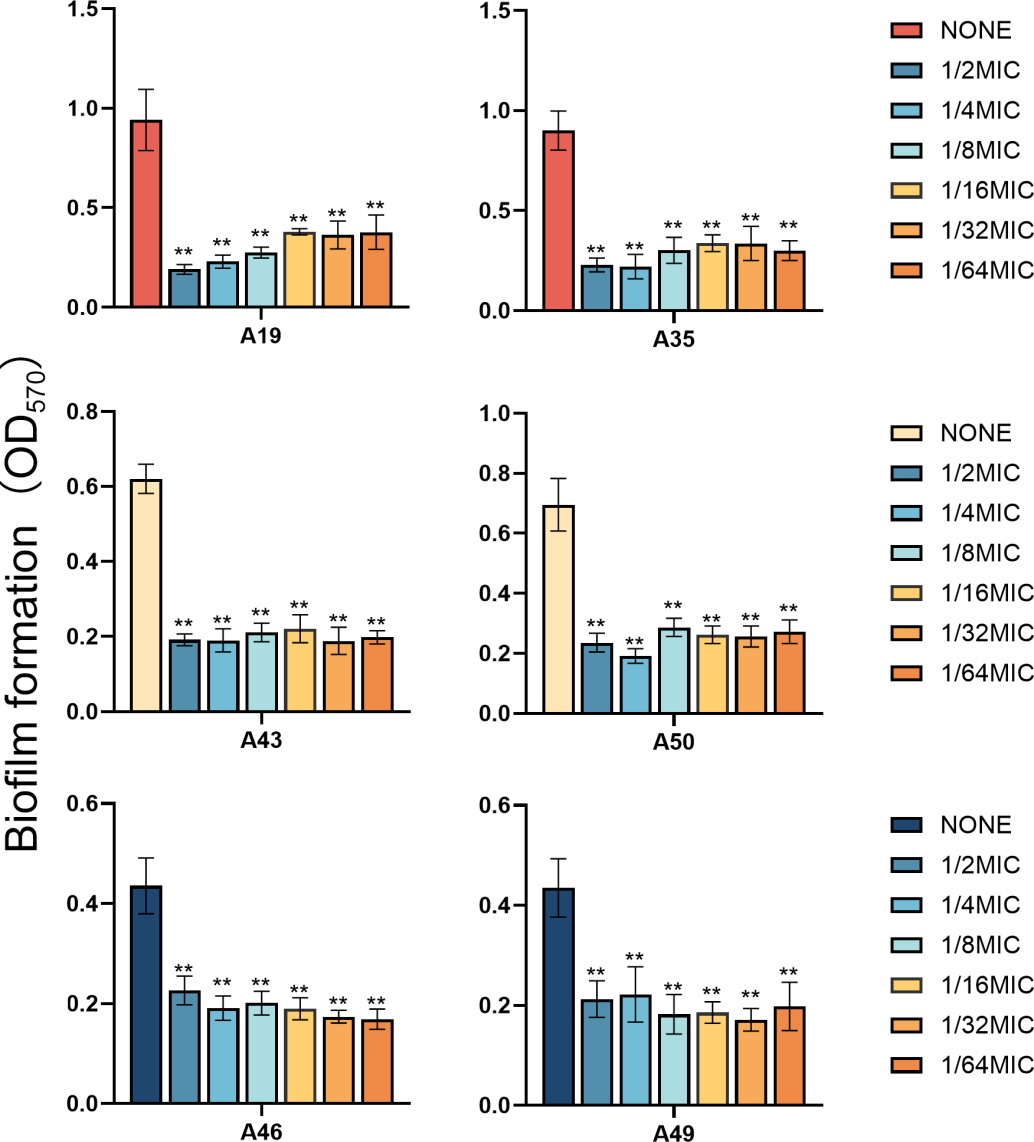
Biofilm formation inhibition of six XDRAB isolates (**A19, A35, A43, A50, A46, and A49**) by 7-hydroxyindole at sub-MIC levels (*n* = 6, $\overset{-}{x}$ ± *s*; **P < 0.01 versus none group).

**Fig 6:**
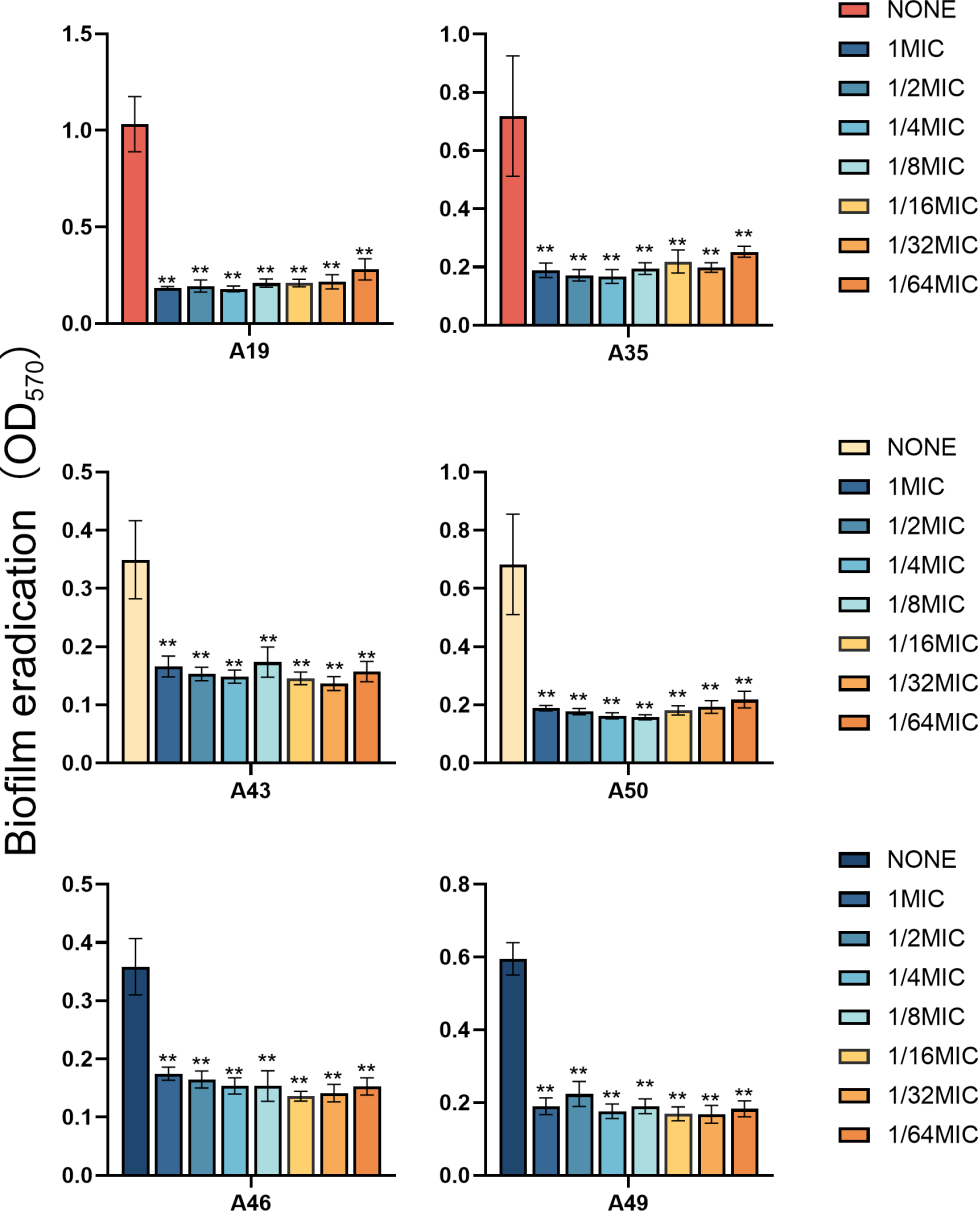
Biofilm eradication of six XDRAB isolates (**A19, A35, A43, A50, A46, and A49**) by 7-hydroxyindole at sub-MIC levels (*n* = 6, $\overset{-}{x}$ ± *s*; **P < 0.01 versus none group).

Because of activities against planktonic cells of XDRAB isolates (Table 1), three indole derivatives—5-iodoindole, 3-methylindole, and 7-hydroxyindole—were further tested for their effects on XDRAB biofilms. Three indoles at 1/2 MIC and 1/4 MIC were found to inhibit XDRAB biofilm formation, with 7-hydroxyindole demonstrating the most potent effect (Fig. 3). Similarly, these indoles at 1× MIC, 1/2 MIC, and 1/4 MIC were also shown to significantly eradicate XDRAB mature biofilms. Intriguingly, at various concentrations tested, strong effects were often evident (Fig. 4). The latter observation led us to further reduce sub-MIC levels of 7-hydroxyindole for the impact on both the inhibition of biofilm formation and the eradication of matured biofilm. Even at a reduced concentration of 1/64 MIC (i.e., 8 µg/mL), 7-hydroxyindole significantly reduced XDRAB biofilm formation (Fig. 5), and notably, it was also able to effectively eradicate XDRAB mature biofilms (Fig. 6).

### Effects of 7-hydroxyindole on XDRAB biofilm assessed using ordinary optical microscope, confocal laser scanning microscopy, and scanning electron microscopy

**Fig 7:**
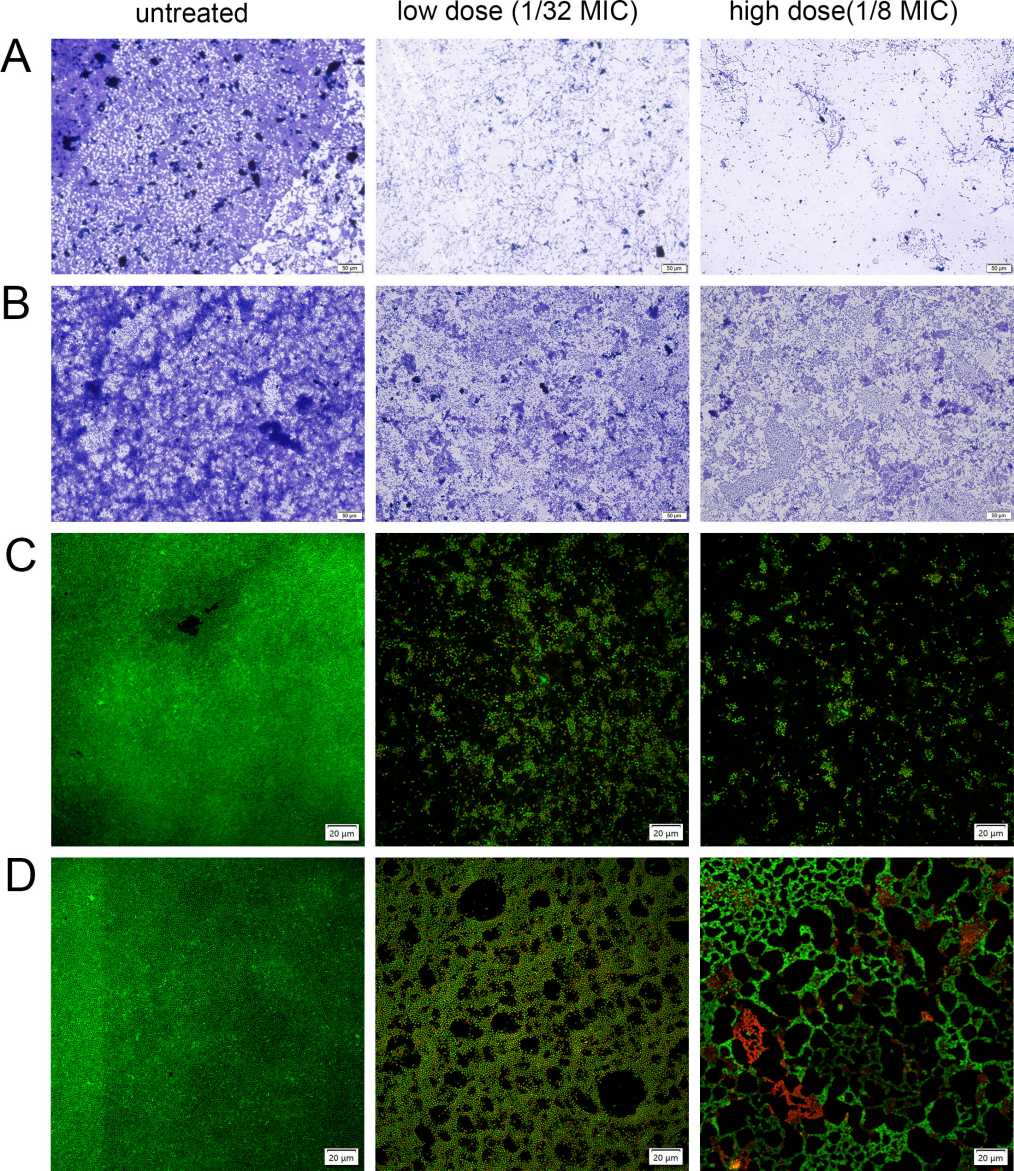
Effects of 7-hydroxyindole at sub-MIC levels (1/32 and 1/64 MIC) on the biofilm of XDRAB isolate A19. The inhibitory effect on XDRAB biofilm in the planktonic state (**A and C**) and the eradication effect on XDRAB biofilm in the encapsulated state (**B and D**) were observed using conventional optical microscopy (A and B; scale bar = 50 µm) and confocal laser scanning microscopy (C and D; scale bar = 20 µm).

To further investigate the effects of 7-hydroxyindole on biofilms, we selected high (1/8 MIC [64 µg/mL]) and low (1/32 MIC [16 µg/mL]) dose levels to treat XDRAB isolate A19 in both planktonic and biofilm states. Observations under an optical microscope revealed that isolate A19 in the planktonic state formed a dense biofilm structure on the cell slide. Upon the addition of 7-hydroxyindole at 1/64 MIC, biofilm formation was significantly reduced. When the concentration was increased to 1/8 MIC, biofilm formation was further diminished, demonstrating a concentration-dependent inhibitory effect (Fig. 7A). At both concentrations, treatment with 7-hydroxyindole in the biofilm state also resulted in a significant eradication in preformed biofilm, and the eradicative effect was similarly concentration-dependent (Fig. 7B).

Confocal laser scanning microscopy (CLSM) further confirmed that 7-hydroxyindole significantly inhibits the formation of isolate A19 biofilm in the planktonic state, resulting in a notably sparser biofilm density compared to the untreated group (Fig. 7C). In the biofilm removal experiments for isolate A19, the low dose of 7-hydroxyindole induced visible holes in the dense biofilm structure; this effect was more pronounced at the higher concentration. These findings suggest that 7-hydroxyindole effectively disrupted isolate A19 mature biofilms, with the eradication effect being concentration-dependent (Fig. 7D).

**Fig 8:**
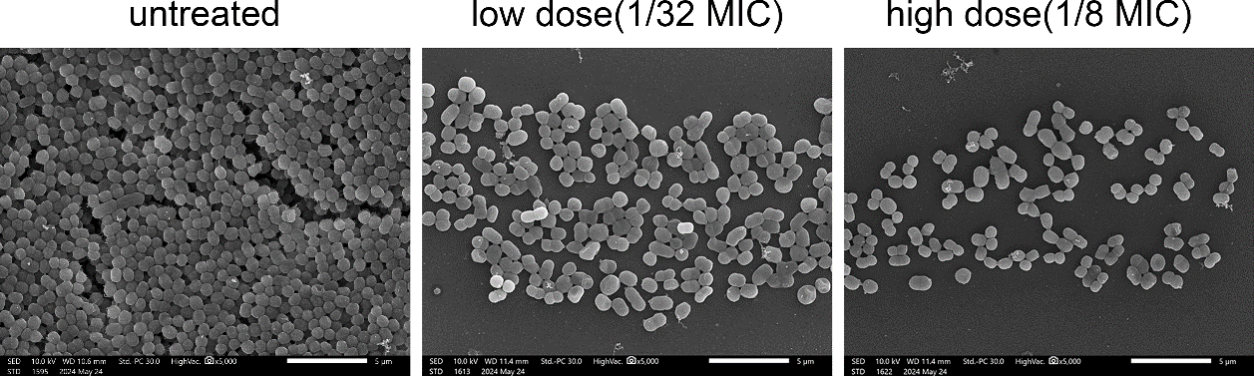
SEM observations of biofilm inhibition of XDRAB isolate A19 by 7-hydroxyindole (scale bar = 5 µm).

The scanning electron microscopy (SEM) analysis revealed that the untreated XDRAB isolate A19 had a high number of biofilm cells that were densely packed, whereas the number of the biofilm cells was significantly reduced following treatment with 7-hydroxyindole, demonstrating a concentration-dependent effect (Fig. 8).

### 7-Hydroxyindole increases the survival of *Galleria mellonella* infected with XDRAB

**Fig 9:**
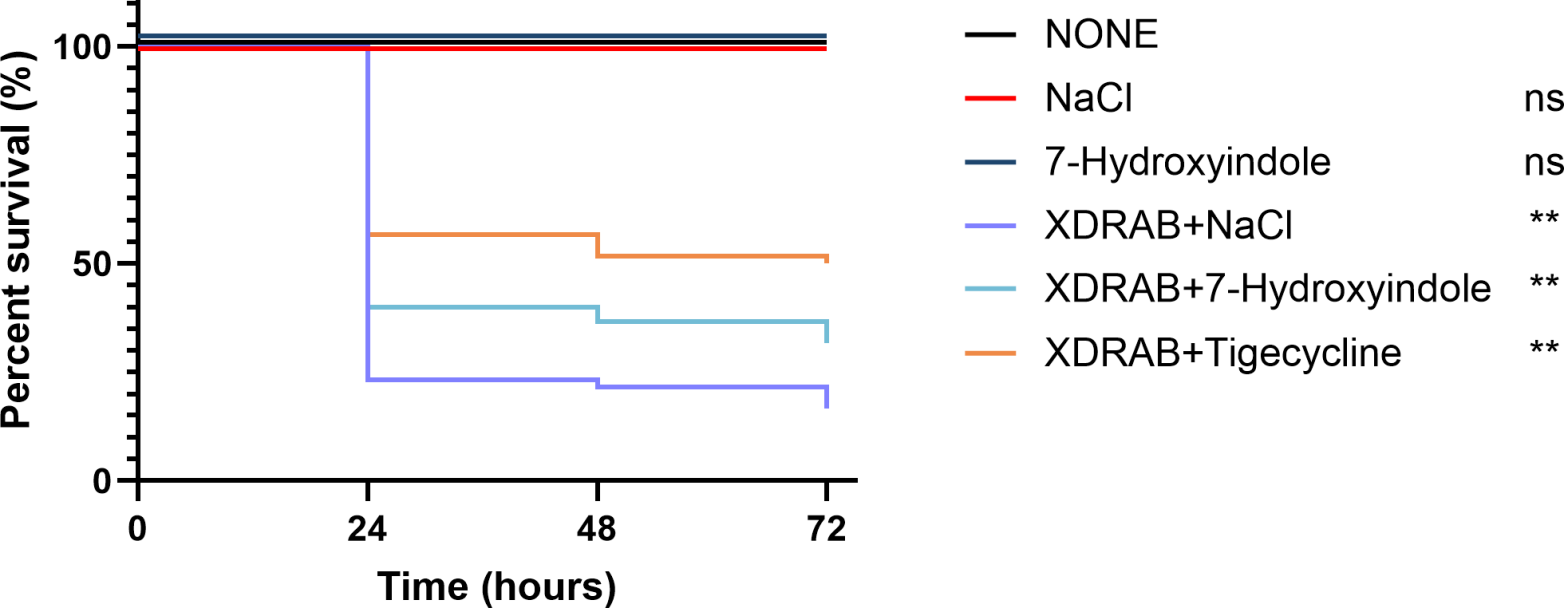
Effect of 7-hydroxyindole on the survival of *G. mellonella* infected with six XDRAB isolates (*n* = 60 per XDRAB-infected group with 10 per isolate; 10 per group without XDRAB inoculation). Compared to the untreated group (none): ns (no statistical significance), *P* > 0.5, **P < 0.01.

To determine the effect of 7-hydroxyindole treatment on the virulence of XDRAB, a *G. mellonella* infection model was used, and the number of survivors of the different treatment groups was recorded for 72 hours. The six XDRAB isolates (A19, A35, A43, A46, A49, and A50) were each used to infect *G. mellonella*. The results showed that after 7-hydroxyindole treatment, the survival rate of *G. mellonella* increased from 16.67% of the untreated group (XDRAB + NaCl) to 31.67% of the treated group (XDRAB + 7-hydroxyindole) (Fig. 9), but was inferior to the tigecycline-treated group (50.00%). The results were consistent with the *in vitro* observations on antimicrobial and antibiofilm activity of 7-hydroxyindole against XDRAB, resulting in an increased survival rate of *G. mellonella*.

### 7-Hydroxyindole affects the expression of quorum sensing-related genes

**Fig 10:**
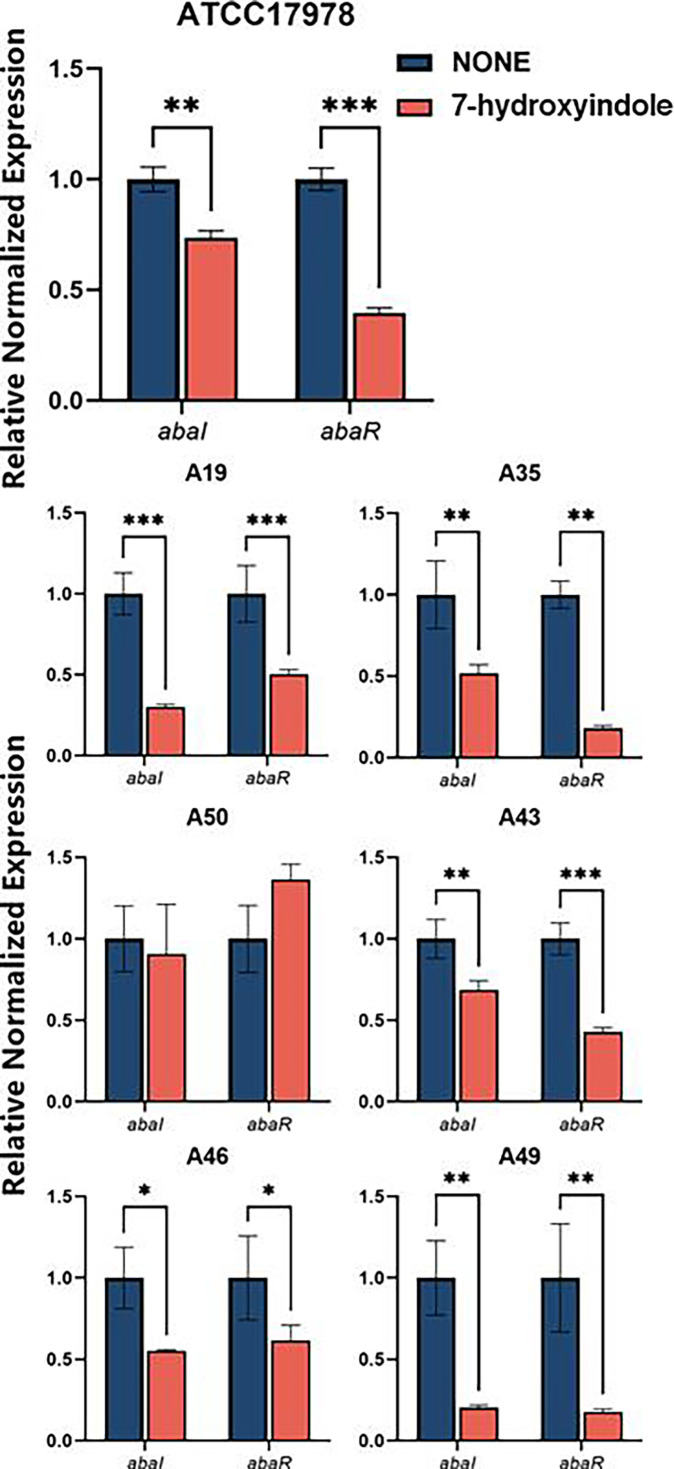
Effect of 7-hydroxyindole on the expression of *abaI* and *abaR* of *A. baumannii* ATCC 17978 and six XDRAB isolates (for each strain, *n* = 6, $\overset{-}{x}$ ± *s*; *P < 0.05, **P < 0.01, ***P < 0.001 versus none group).

To explore the mechanism by which 7-hydroxyindole inhibits XDRAB biofilm formation, we examined its effects on the expression of quorum sensing-related genes affecting *A. baumannii* biofilm formation (38, 39). Reverse transcription (RT)-qPCR was employed to assess the impact of 7-hydroxyindole on the expression of *abaI* and *abaR* of six XDRAB isolates and the reference strain ATCC17978. The two genes encode, respectively, a quorum sensing system auto-inducer synthase (AbaI) and an auto-inducer synthase receptor (AbaR) involved in biofilm formation and virulence (38). The findings indicated that 7-hydroxyindole significantly inhibited both *abaI* and *abaR* expression at 1/8 MIC in all tested strains, except for strain A50 where the *abaI* and *abaR* expression were moderately down-regulated and up-regulated, respectively (Fig. 10).

## DISCUSSION

XDRAB isolates are frequently prevalent in clinical settings around the globe and pose a major threat to effective antimicrobial therapy (4, 7, 10, 40, 41). In a recent national cohort of patients with XDRAB, neither combination therapy nor receipt of adequate treatment was found to improve outcomes, indicating the need for optimal management of this difficult-to-treat pathogen with few effective antimicrobial options (42). Indeed, *A. baumannii* is featured with multidrug and extensive drug resistance and with its adhesion to medical devices (e.g., catheters, endotracheal tubes of ventilators, dialysis equipment, and various surfaces) by forming biofilms (43, 44). These features, together with antimicrobial use, are major risk factors related to XDRAB nosocomial infections or outbreaks (44–46). Consequently, antimicrobial activity against XDRAB and inhibiting XDRAB biofilm formation are crucial for controlling XDRAB infections. This study revealed the high prevalence of XDRAB (including carbapenem resistance) in our clinical setting (Table 1 and Table S3), consistent with global increasing trends regarding resistance in *A. baumannii*, which is highlighted by the World Health Organization and US Centers for Disease Control and Prevention as a major public health threat (9, 47). Over 90% of the 70 isolates in this study formed various degrees of biofilms, which aligns with the higher rate of biofilm production in *A. baumannii* (44).

Given that indole derivatives possess potential antimicrobial activities (22, 23, 25, 34), we initiated an investigation to screen 46 indole agents for their antimicrobial and antibiofilm activities. These agents showed MIC values of 64–1,024 µg/mL. Subsequently, we further focused on three indole derivatives: 5-iodoindole, 3-methylindole, and particularly, 7-hydroxyindole. These three indoles exhibit relatively strong activity against XDRAB isolates and also interplay with anti-*A*. *baumannii* antimicrobial drugs for synergistic effects. These agents at sub-MIC levels inhibited XDRAB biofilm formation. More importantly, 7-hydroxyindole was able to eradicate established biofilms.

The intrinsic antimicrobial activity of indole derivatives is expected based on available literature information (22, 48). For instance, a range of *bis*-indole agents was found to exhibit antimicrobial activity against multidrug-resistant gram-positive and gram-negative bacterial species, including *A. baumannii* and *P. aeruginosa* (29, 33, 34, 49). Our observations of the synergistic effects of indole agent-antimicrobial drug combinations reflect well the interplay between indole agents and conventional antimicrobial drugs (29, 49). Another study also showed that 3-indoleacetonitrile enhanced susceptibility to imipenem and attenuated biofilm formation in *A. baumannii* (50). The indole derivatives are warranted for further investigations regarding their potential to be used at sub-inhibitory levels as antimicrobial adjuvants. Furthermore, the eradicating effects on XRAB mature biofilm, as demonstrated with sub-inhibitory levels of 7-hydroxyindole, indicate another important property of indole agents against gram-negative bacterial biofilm cells.

We further explored potential mechanisms related to the antimicrobial and/or antibiofilm activities of indole agents. First, antimicrobial activities of indole agents are predicted to be possibly attributable to multiple modes of action, including DNA targeting and membrane integrity disruption (51, 52). The mechanisms of action of indole agents are mostly different from those of conventional antimicrobial drugs (53), thus allowing indoles to inhibit or kill XDRAB. Indoles can facilitate the uptake of antimicrobials in gram-negative bacteria and provide support for combination antimicrobial regimens (50, 54). Intriguingly, a recent study on the structure-activity relation of an indole agent revealed the influence of indole substitution at positions 5 and 7 on their biological activity (55). These substitute positions are reflected in this study with the different groups of our two selected indole derivatives (5-iodoindole and 7-hydroxyindole). Biofilm formation in *A. baumannii* is multifactorial, including contributions from the quorum sensing system (39, 56). In this regard, AbaI/AbaR form key components of the *A. baumannii* quorum sensing system and are homologs of LuxI/LuxR widely present in other gram-negative bacteria (38, 57). Our results indicate that indoles significantly inhibited the expression of *abaI* and *abaR*, likely suggesting that a possible mechanism by which indole agents inhibit or eradicate XDRAB biofilms may at least involve disrupting the quorum sensing pathways of *A. baumannii*. Moreover, we consider that indole agents may be potentially used as biocides/disinfectants, which have demonstrated activities against *A. baumannii* including biofilms (58, 59). In this regard, multidrug efflux pumps of the resistance-nodulation-cell division (RND) superfamily in *A. baumannii* play a major role in intrinsic and acquired multidrug resistance (6). Unlike various conventional antimicrobial drugs, biocides may not be preferred substrates for RND pumps (6, 60). However, biocide agents may affect the expression of RND pumps in *A. baumannii* as part of the bacterial stress response to bioactive agents (60). Migliaccio et al. (58) recently demonstrated modulation of efflux pump activity by resveratrol, chlorhexidine, and benzalkonium for inhibition of biofilm formation and preformed biofilm in *A. baumannii*. Thus, whether and how indole agents presented in this study could affect the expression of RND and other family drug efflux pumps is warranted for further investigation. Together, our study supports the potential for further exploring indole agents as antimicrobials, biocides, or adjuvants in combating antimicrobial-resistant pathogens, including the increasing threats associated with XDRAB.

## MATERIALS AND METHODS

### Bacterial strains, growth media, and antimicrobial agents

A total of 70 clinical isolates of *A. baumannii* were obtained from clinical specimens in 2018–2019 from the First Affiliated Hospital of Chengdu Medical College, Chengdu, China (Tables S2 and S3). These isolates were assessed for their abilities to form biofilms (Table S4). Six isolates (i.e., A19, A35, A43, A46, A49, and A50) with various degrees (strong, medium, and weak, respectively, two isolates from each category) were selected for assessing antimicrobial and antibiofilm effects of indole derivative agents. These isolates were identified to belong to sequence type ST 298 except isolate A49 as ST 195 based on multilocus sequence typing analysis as previously described (59) from genomic sequences of seven housekeeping genes *gltA*, *gyrB*, *gdhB*, *recA*, *cpn60*, *gpi*, and *rpoD* (61, 62). Studies have shown that the ability to form biofilm differs among *A. baumannii* isolates assigned to distinct genotypes (10). *A. baumannii* ATCC 17978, a reference strain (63, 64), was used for gene expression testing. *E. coli* ATCC 25922 and *Staphylococcus aureus* ATCC 29213 are quality control strains for antimicrobial susceptibility testing (37).

Bacterial cells were cultured in Luria Bertani (LB) broth, LB agar medium, tryptone soy broth (TSB) medium, or cation-adjusted Muller-Hinton broth (CAMHB) as described in relevant experiments. Clinically relevant antimicrobials used for susceptibility testing include β-lactams (meropenem, imipenem, cefotaxime, ceftazidime, ampicillin-sulbactam, and cefoperazone-sulbactam), aminoglycosides (amikacin and gentamicin), fluoroquinolones (ciprofloxacin and levofloxacin), polymyxin (polymyxin B), and tetracyclines (doxycycline, minocycline, tetracycline, and tigecycline). A total of 46 indole agents, including 5-iodoindole, 3-methylindole, and 7-hydroxyindole, were tested. Details of bacterial strains, media, and chemicals (including indole agents and antimicrobial drugs) are present in Table S1.

### Antimicrobial and indole agent susceptibility testing

The MIC values of antimicrobial drugs and indole derivatives against *A. baumannii* were determined by the broth microdilution method (37). Briefly, bacterial cells were grown on LB agar medium overnight at 37°C, subsequently resuspended in physiological saline to be adjusted to the 0.5 McFarland turbidity standard and then diluted 20 times. Antimicrobial drugs were prepared in water, and indole derivatives were dissolved in dimethyl sulfoxide. Stock solutions of these agents were then diluted to different concentrations using CAMHB medium. Finally, 180 µL of CAMHB, 10 µL of the corresponding antimicrobial drug or an indole derivative, and 10 µL of bacterial resuspension were added to the 96-well plate. Optical density at 600 nm (OD_600_) was measured after incubation at 37°C in the dark for 20–24 hours. An OD_600_ value of less than 0.1 was regarded as no bacterial growth, colored drugs were observed macroscopically, and data were recorded.

### Antimicrobial synergy testing

The checkerboard method was applied to assess the synergistic effects of three indole derivatives and conventional antimicrobial drugs on six XDRAB. Similar to antimicrobial susceptibility testing described above, each plate well in this combination testing contained 170 µL CAMHB, 10 µL of serially diluted indole derivatives along the *x*-axis, 10 µL of antimicrobial along the *y*-axis, and 10 µL of bacterial resuspension, followed by incubation at 37°C for 20–24 hours. The fractional inhibitory concentration index (FICI) values were obtained by the following equation:


$$FICI=\frac{MIC_{A\;Combination}}{MIC_{A\;alone}}+\frac{MIC_{B\;Combination}}{MIC_{B\;alone}}$$

Interpretation of the FICI values was as follows: synergy (FICI ≤ 0.5), no interaction (FICI > 0.5–4.0) (FICI ≤ 0.5), and antagonism (FICI > 4.0) (65). Within no interaction, additivity (>0.5–1) and indifference (>1–4) were determined.

### Biofilm formation of XDRAB and effects of indole derivatives

A crystal violet staining method was used to determine the biofilm formation ability of XDRAB and the effects of indole derivatives (66, 67). Initially, 70 isolates of *A. baumannii* were screened for their biofilm formation abilities, followed by grouping them as strong (OD_570_ ≥ 3OD_570_ [Blank] + 3SD), medium (3OD_570_ [Blank] + 3SD > OD_570_ ≥ 2OD_570_ [Blank] + 3SD), and weak (OD_570_ ≥ OD_570_ [Blank] + 3SD), biofilm producers (67). Briefly, bacterial cells grown overnight at 37°C were adjusted to 0.5 McFarland turbidity standard. Using a 96-well cell culture plate, 170 µL of TSB medium and 10 µL of phosphate-buffered saline (PBS; no treatment control group) or different concentrations of an indole derivative solution were added to each well, followed by the inoculation of 20 µL of the bacterial suspension. To avoid the edge effect of the 96-well plate, the surrounding wells were removed, and five replicate wells were set for each strain. The culture plate was incubated at 37°C for 24 hours, and then the wells were emptied and washed three times with PBS. After complete drying, 150 µL of 0.5% crystal violet was added for 20 minutes, then washed three times with PBS, dried in air for 30 minutes, dissolved in 150 µL of 95% ethanol for 15 minutes, and finally OD_570_ values were measured to quantify the amount of XDAB biofilm formation

For assessing the biofilm eradication effect, 180 µL of TSB medium and 20 µL of bacterial solution were added, followed by incubation at 37°C for 24 hours. After the biofilm was formed, the wells were emptied and washed three times with PBS. Subsequently, 190 µL PBS and 10 µL of indole derivative solutions were added and cultured at 37°C for 24 hours. Finally, crystal violet staining and OD_570_ determination were performed.

### *Galleria mellonella* killing assay

*G. mellonella* serves as a model system to examine *A. baumannii* pathogenesis (68). *G. mellonella* infection assay was conducted to assess the impact of 7-hydroxyindole on the killing by the six XDRAB isolates. Ten larvae per group or per isolate were randomly allocated. *G. mellonella* larvae with a length of approximately 15–25 mm and a weight ranging from 250 to 350 mg were selected for the experiment, and injections were administered via a microsyringe into the penultimate right hind leg. Each larva was infected with 10 µL of a bacterial suspension containing approximately 2.5 × 10^6^ colony-forming units of XDRAB. After 30 minutes of infection, the treatment group was injected with 7-hydroxyindole (10 µL at 1× MIC). The control groups included an untreated group (received 0.9% sodium chloride) and an antimicrobial treated group (10 µL tigecycline at 1× MIC). The treated larvae were reared in a constant temperature incubator set at 37℃, followed by the examination of the mortality of *G. mellonella* larvae every 24 hours post-treatment for 72 hours.

### Effect of 7-hydroxyindole on XDRAB biofilm assessed using ordinary optical microscope, CLSM, and SEM

Cell slides with surface tissue culture treated were added to 24-well plates, followed by the addition of 850 µL of TSB medium, 100 µL of 0.5 McFarland turbidity standard bacterial solution, and 50 µL of 7-hydroxyindole (high dose: 1/8 MIC; low dose: 1/32 MIC; and 50 µL of 0.125% dimethylsulfoxide as the blank control). The culture plate was incubated at 37°C for 24 hours, which allowed the strain to grow, adhere to the cell slide, and form biofilms. Subsequently, the wells were emptied and washed three times with PBS. In addition, for the detection of the eradication effect, only 900 µL TSB medium and 100 µL of the above bacterial solution were added and cultured at 37°C for 24 hours. Then, 950 µL PBS and 50 µL of different concentrations of indole derivative solution were added and cultured at 37°C for 24 hours. For the conventional light microscopy examination, after washing away the floating bacterial cells, the biofilm cells were fixed with 2.5% glutaraldehyde for 20 minutes, stained with 0.5% crystal violet for 15 minutes, and finally, washed three times with PBS and observed with a conventional light microscope. For CLSM examination, SYTO 9 (5 μM) and propidium iodide (10 µM) dyes were used to stain bacterial cells for 15 minutes, followed by stain removal, washing with PBS, and slide preparation. For SEM examination, bacterial cells were fixed with 2.5% glutaraldehyde overnight at 4°C, followed by dehydration in graded eight ethanol solutions (30%–100%; 15 minutes each). Finally, biofilm specimens were dried and observed using SEM imaging (69, 70).

### Gene expression assay

RT-qPCR was used to assess the effect of 7-hydroxyindole on the gene expression of quorum sensing/biofilm formation genes (*abaI* and *abaR*) of *A. baumannii* strains. The primers used are presented in Table S6. Briefly, 50 µL of XDRAB cells (0.5 McFarland) and 50 µL of 7-hydroxyindole at different concentrations were, respectively, added to 900 µL LB broth, followed by incubation at 37°C for 24 hours. Total RNA was prepared using an RNA isolation kit (Vazyme Biological, Nanjing, China) from *A. baumannii*, followed by RT-qPCR that included the reverse transcription of RNA into cDNA using a cDNA synthesis kit (Vazyme) and qPCR using the fluorescent dye SYBR Color qPCR Master Mix (Vazyme). The 16S rRNA gene was used as the internal standard of mRNA quantification. The qPCR cycling conditions were as follows: pre-denaturation at 95°C for 3 minutes, followed by 40 cycles of 95°C for 15 seconds and 60°C for 30 seconds. The expression level of each gene was normalized, and the relative expression was calculated as 2^−ΔΔCT^. Fold changes in gene expression from 7-hydroxyindole-treated cells were compared to untreated cells that were propagated under the same conditions (71).

### Statistical analysis

The mean and standard deviation of the mean were calculated using SPSS 27.0. GraphPad Prism 10.2.3 was used for mapping (https://www.graphpad.com/). Data were analyzed with a one-way analysis of variance followed by Dunnett’s test. Data were considered statistically significant with *P* < 0.05.

## SUPPLEMENTAL MATERIAL

Supplemental material spectrum.03388-24-s0001.docx Tables S1 to S6.

ASM does not own the copyrights to Supplemental Material that may be linked to, or accessed through, an article. The authors have granted ASM a non-exclusive, world-wide license to publish the Supplemental Material files. Please contact the corresponding author directly for reuse.
