# Supplementary material for: Indole derivatives display antimicrobial and antibiofilm effects against extensively drug-resistant Acinetobacter baumannii
Source: Microbiol Spectr. 2025 Apr 15;13(5):e03388-24. doi: 10.1128/spectrum.03388-24 (PMC12073863; doi:10.1128/spectrum.03388-24)
Supplement: Supplemental material — Tables S1 to S6. [file spectrum.03388-24-s0001.docx]

**Supplemental Materials**

**TABLE S1** Bacterial strains, media and chemicals (including indole agents and antimicrobial drugs)

| Reagents | Source | Identifier |
| --- | --- | --- |
| *Bacterial strains* |  |  |
| *Acinetobacter baumannii* | ATCC, Manassas, VA, USA | ATCC 17978 |
| *Escherichia coli* | ATCC, Manassas, VA, USA | ATCC 25922 |
| *Staphylococcus aureus* | ATCC,  Manassas, VA, USA | ATCC 29213 |
| Extensively drug-resistant *A. baumannii* | First Affiliated Hospital of Chengdu Medical College | A19, A35, A43, A46, A49, and A50 |
| *Bacterial culture media* | | |
| LB broth | Hopebio | Cat# HB0128 |
| CAMHB | Hopebio | Cat# HB6231-1 |
| TSB | Hopebio | Cat# HB4114 |
| *Chemicals (including indole derivatives and antimicrobial drugs)* | | |
| 5-Iodoindole | Macklin | Cat# I812160 |
| 5-Fluoroindole | Macklin | Cat# F809692 |
| 6-Bromoindole | Macklin | Cat# B803970 |
| 6-Iodoindole | Macklin | Cat# I893099 |
| 5-Nitroindole | Macklin | Cat# N814694 |
| 5,6-Dihydroxyindole | Macklin | Cat# D843124 |
| 7-Chloroindole | Macklin | Cat# C805791 |
| 4-Bromoindole | Macklin | Cat# B802829 |
| 7-Fluoroindole | Macklin | Cat# F809690 |
| 5-Methylindole | Macklin | Cat# M813747 |
| 6-Methylindole | Macklin | Cat# M813358 |
| 3-Methylindoline | Macklin | Cat# M932891 |
| Indigo red | Macklin | Cat# I811709 |
| 4-Methylindole | Macklin | Cat# M813360 |
| 2-Methylindoline | Macklin | Cat# M813043 |
| 7-Hydroxyindole | Macklin | Cat# H825113 |
| 4-Hydroxyindole | Macklin | Cat# H810933 |
| Indole | Macklin | Cat# I811715 |
| Melatonin | MeilunBio | Cat# MB1475 |
| Indole-3-acetate methyl ester | Macklin | Cat# M824030 |
| 3-Indoleacetonitrile | Macklin | Cat# I811818 |
| DL-5-Hydroxytryptophan | Macklin | Cat# H811204 |
| 1H-Indole-3-sulfonate potassium salt | Titan | Cat# Y43038A |
| 3-Methylindole | Macklin | Cat# S817593 |
| 4-Chloroindole | Macklin | Cat# C804912 |
| 5-Bromoindole | Macklin | Cat# B802828 |
| 4-Iodoindole | Bidepharm | Cat# BD227965 |
| 7-Nitroindole | Macklin | Cat# N814814 |
| 4-Fluoroindole | Macklin | Cat# F809693 |
| 6-Chloroindole | Macklin | Cat# C806182 |
| 5-Fluoroindole | Macklin | Cat# F809692 |
| 7-Iodoindole | Bidepharm | Cat# BD221314 |
| 3,3'-Methylenediindole | Macklin | Cat# D830949 |
| 7-Methylindole | Macklin | Cat# M813359 |
| 5-Trifluoromethoxyindole-2,3-dione | Macklin | Cat# T826144 |
| 3-Indolecarboxaldehyde | Macklin | Cat# I811783 |
| 2-Methylindole | Macklin | Cat# M812752 |
| 5-Iododihydroindole-2,3-dione | Macklin | Cat# BD5663 |
| 5-Hydroxyindole | Macklin | Cat# H810947 |
| 6-Hydroxyindole | Macklin | Cat# H810822 |
| 1-Methylindole | Macklin | Cat# M813366 |
| 3-Indoleacetic acid | Macklin | Cat# I811602 |
| Indigo blue | MeilunBio | Cat# MB4677 |
| 3-Indolepropionic acid | Macklin | Cat# I811687 |
| Midostaurin | MedMol | Cat# S80167 |
| Vincamine | Macklin | Cat# V820436 |
| Dimethylsulfoxide | HuShi | Cat#30072418 |
| Crystal violet | MeilunBio | Cat# MB4721 |
| Ampicillin | Sangon Biotech | Cat# A610029-0025 |
| Ceftazidime | MeilunBio | Cat# MB1334 |
| Cefotaxime | MeilunBio | Cat# MB5528 |
| Ampicillin-sulbactam (2:1) | Sichuan Pharmaceutical Preparations | Not available |
| Cefoperazone-sulbactam (2:1) | Pfizer | Not available |
| Imipenem | MeilunBio | Cat# MB1457 |
| Meropenem | MeilunBio | Cat# MB1129 |
| Amikacin | MeilunBio | Cat# MB1012 |
| Gentamicin | MeilunBio | Cat# MB1331 |
| Ciprofloxacin | MeilunBio | Cat# MB1283 |
| Levofloxacin | MeilunBio | Cat# MB1576-1 |
| Tetracycline | MeilunBio | Cat# MB2004 |
| Doxycycline | MeilunBio | Cat# MB1088 |
| Tigecycline | MeilunBio | Cat# MB1246 |
| Polymyxin **B** | MeilunBio | Cat# MB1188 |

**TABLE S2** Antimicrobial susceptibility of 70 clinical isolates of *Acinetobacter baumannii*

| Strain | Imipenem | Meropenem | Ceftazidime | Ceftizoxime | Cefoperazone-Sulbactam (2:1) | Ampicillin | Amikacin | Gentamicin | Ciprofloxacin | Levofloxacin | Tetracycline | Doxycycline | Minocycline | Tigecycline | Azithromycin | Polymyxin B | Phenotype* |
| --- | --- | --- | --- | --- | --- | --- | --- | --- | --- | --- | --- | --- | --- | --- | --- | --- | --- |
| A1 | 32 | 32 | 1024 | 128 | 256 | ˃1024 | ˃1024 | ˃1024 | 64 | 8 | 512 | 32 | 16 | 8 | 0.5 | 2 | XDR |
| A2 | 64 | 32 | ˃ 1024 | 128 | 128 | ˃1024 | ˃1024 | ˃1024 | 256 | 8 | 1024 | 64 | 8 | 1024 | 0.5 | 0.25 | XDR |
| A3 | 64 | 64 | >1024 | >1024 | 512 | ˃1024 | 256 | >1024 | 128 | 8 | 512 | 32 | 8 | ˃ 1024 | 1 | 2 | XDR |
| A4 | 32 | 16 | 256 | 128 | 128 | ˃1024 | ˃1024 | 16 | 64 | 2 | 512 | 32 | 2 | 4 | 0.5 | 1 | MDR |
| A5 | 32 | 32 | >1024 | 256 | 64 | ˃1024 | ˃1024 | ˃1025 | 32 | 8 | 512 | 64 | 4 | 256 | 0.5 | 2 | XDR |
| A6 | 64 | 64 | >1024 | 64 | 256 | ˃1024 | ˃1024 | ˃1024 | 64 | 16 | 512 | 128 | 8 | 1024 | 1 | 0.25 | XDR |
| A7 | 32 | 64 | 256 | 128 | 256 | ˃1024 | ˃1024 | ˃1024 | 128 | 16 | 512 | ˃256 | 8 | 1024 | 0.5 | 4 | XDR |
| A8 | 64 | 32 | 256 | 128 | 128 | ˃1024 | ˃1024 | ˃1024 | 64 | 16 | 512 | ˃256 | 8 | 1024 | 1 | 4 | XDR |
| A9 | 32 | 16 | >1024 | 128 | 128 | ˃1024 | ˃1024 | >1025 | 64 | 4 | 1024 | 128 | 8 | 1024 | 1 | 1 | XDR |
| A10 | 16 | 16 | >1024 | 64 | 128 | ˃1024 | ˃1024 | ˃1024 | 128 | 16 | 512 | 64 | 32 | 1024 | 0.25 | 4 | XDR |
| A11 | 16 | 32 | >1024 | >128 | 256 | ˃1024 | 256 | >1024 | 128 | 8 | 512 | 64 | 32 | 512 | 0.5 | 4 | XDR |
| A12 | 64 | 32 | 128 | 128 | 128 | ˃1024 | ˃1024 | ˃1024 | 64 | 16 | 512 | 64 | 8 | 1024 | 0.5 | 0.25 | XDR |
| A13 | 32 | 64 | >1024 | 128 | 256 | ˃ 1024 | ˃1024 | ˃1024 | 128 | 16 | 512 | 64 | 32 | 4 | 0.5 | 4 | XDR |
| A14 | 32 | 32 | 128 | 128 | 256 | ˃1024 | ˃1024 | ˃1024 | 64 | 16 | 512 | ˃256 | 8 | ˃ 1024 | 0.5 | 2 | XDR |
| A15 | 128 | 64 | 256 | 128 | 512 | ˃1024 | ˃1024 | ˃1024 | 64 | 8 | 512 | ˃256 | 8 | 1024 | 0.25 | 2 | XDR |
| A16 | 2 | 16 | 256 | 512 | 32 | ˃1024 | ˃1024 | ˃1024 | 256 | 32 | 512 | ˃256 | 16 | ˃ 1024 | 0.5 | 4 | MDR |
| A17 | 64 | 64 | 256 | 128 | 256 | ˃1024 | ˃1024 | ˃1024 | 64 | 16 | 512 | ˃256 | 8 | 512 | 0.5 | 0.25 | XDR |
| A18 | 16 | 8 | 512 | 512 | 32 | ˃1024 | ˃1024 | 256 | 16 | 8 | 512 | 128 | 4 | 1024 | 0.5 | 2 | MDR |
| A19 | 32 | 16 | >1024 | 128 | 256 | ˃1024 | ˃1024 | ˃1024 | 64 | 16 | 512 | 64 | 16 | 256 | 0.5 | 4 | XDR |
| A20 | <4 | <4 | <8 | <8 | <16 | 64 | <8 | <4 | <1 | <2 | <4 | <16 | <4 | <4 | 0.5 | 2 | S |
| A21 | 64 | 64 | 128 | 128 | 512 | ˃1024 | ˃1024 | ˃1024 | 64 | 16 | 512 | 64 | 8 | ˃ 512 | 1 | <0.125 | XDR |
| A22 | 32 | 64 | >1024 | 128 | 128 | ˃10248 | 512 | ˃1024 | 64 | 16 | 512 | 64 | 8 | 1024 | 0.5 | >4 | XDR |
| A23 | 64 | 64 | >1024 | 64 | 256 | >1024 | ˃1024 | ˃1024 | 128 | 8 | 256 | 64 | 16 | ˃ 1024 | 1 | 4 | XDR |
| A24 | 128 | 64 | 512 | 64 | 512 | >1024 | ˃1024 | ˃1024 | 16 | 16 | 512 | 64 | 8 | 1024 | 0.5 | 4 | XDR |
| A25 | 16 | 64 | 512 | 64 | 512 | >1024 | ˃1024 | ˃1024 | 16 | 16 | 512 | 64 | 16 | ˃ 1024 | 1 | 2 | XDR |
| A26 | 32 | 32 | >1024 | 128 | 64 | ˃1024 | ˃1024 | ˃1024 | 256 | 16 | 512 | 64 | 8 | ˃ 1024 | 0.5 | 4 | XDR |
| A27 | 2 | 1 | >1024 | 64 | 16 | 1024 | ˃1024 | ˃1024 | 128 | 16 | 256 | 64 | 32 | 1024 | 1 | 4 | MDR |
| A28 | 32 | 128 | >1024 | 128 | 256 | ˃2048 | ˃1024 | ˃1024 | 128 | 8 | 512 | 64 | 8 | 1024 | 0.5 | 4 | XDR |
| A29 | 64 | 64 | 512 | 128 | 512 | ˃1024 | ˃1024 | ˃1024 | 16 | 16 | 512 | 64 | 16 | ˃ 32 | 0.5 | 4 | XDR |
| A30 | <4 | <4 | <8 | <8 | <16 | <32 | <8 | <4 | <1 | <2 | <4 | <4 | <4 | <4 | 0.25 | 4 | S |
| A31 | 16 | 32 | 512 | 128 | 256 | ˃1024 | 512 | >128 | 16 | 16 | 512 | 64 | 16 | 256 | 0.5 | 4 | XDR |
| A32 | 64 | 128 | >1024 | 64 | 256 | ˃2048 | ˃1024 | ˃1024 | 128 | 32 | 512 | 64 | 8 | 256 | 0.5 | 2 | XDR |
| A33 | 16 | 64 | 512 | 128 | 512 | ˃1024 | 512 | ˃1024 | 16 | 16 | 512 | 64 | 16 | ˃ 32 | 0.5 | 4 | XDR |
| A34 | 32 | 64 | 256 | 128 | 128 | ˃1024 | ˃1024 | ˃1024 | 64 | 8 | 512 | 32 | 16 | 256 | 0.5 | 2 | XDR |
| A35 | 32 | 64 | >1024 | 64 | 256 | >1024 | ˃1024 | ˃1024 | 128 | 8 | >1024 | 32 | 8 | 1024 | 0.5 | 8 | XDR |
| A36 | 64 | 32 | 128 | 128 | 128 | ˃1024 | ˃1024 | ˃1024 | 64 | 8 | 512 | 64 | 16 | 256 | 2 | <0.125 | XDR |
| A37 | 4 | 32 | 128 | 64 | 128 | ˃1024 | ˃1024 | ˃1024 | 32 | 8 | 64 | 32 | 8 | ˃ 32 | 0.06 | 4 | XDR |
| A38 | 64 | 64 | 256 | 128 | 128 | ˃1024 | ˃1024 | >1024 | 64 | 8 | ˃1024 | 32 | 16 | 256 | 1 | 4 | XDR |
| A39 | <4 | <4 | <8 | <8 | <16 | <32 | <8 | <4 | <1 | <2 | <4 | <4 | <4 | <4 | 0.25 | 1 | S |
| A40 | <4 | <8 | <8 | <8 | <16 | <32 | <8 | <4 | <1 | <2 | <4 | <4 | <4 | <4 | 0.25 | 0.5 | S |
| A41 | <4 | <8 | <8 | <8 | <16 | <32 | <8 | <4 | <1 | <2 | <4 | <4 | <4 | <4 | 0.25 | 1 | S |
| A42 | <4 | <8 | <8 | <8 | <16 | 64 | <8 | <4 | <1 | <2 | <4 | <4 | <4 | 4 | 0.25 | 1 | S |
| A43 | 32 | 64 | 512 | 64 | 256 | >1024 | 256 | ˃1024 | 16 | 16 | 512 | 64 | 8 | 256 | 0.5 | 4 | XDR |
| A44 | 16 | 32 | 512 | 64 | 128 | ˃1024 | 256 | 512 | 16 | 16 | 512 | 64 | 8 | >32 | 1 | 2 | XDR |
| A45 | 32 | 64 | 512 | 128 | 256 | ˃1024 | 256 | ˃1024 | 16 | 16 | 512 | 64 | 16 | 256 | 0.5 | 2 | XDR |
| A46 | 32 | 64 | >1024 | 128 | 256 | ˃2048 | ˃1024 | ˃1024 | 256 | 16 | 512 | 64 | 8 | 1024 | 0.5 | 1 | XDR |
| A47 | 128 | 64 | >1024 | 128 | 256 | ˃1024 | ˃1024 | ˃1024 | 128 | 8 | 512 | 64 | 8 | 1024 | 0.25 | 0.25 | XDR |
| A48 | 32 | <8 | <16 | <8 | 64 | 32 | <8 | 8 | 128 | <2 | <32 | <4 | <4 | 4 | 1 | 0.5 | MDR |
| A49 | 32 | 16 | >1024 | 1024 | 256 | ˃1024 | ˃1024 | ˃1024 | 64 | 8 | 512 | 32 | 4 | 1024 | 0.25 | 0.25 | XDR |
| A50 | 32 | 64 | >1024 | 128 | 256 | ˃1024 | 32 | ˃1024 | 64 | 8 | 512 | 64 | 8 | 1024 | 1 | 4 | XDR |
| A51 | 16 | 16 | >1024 | 128 | 256 | ˃1024 | ˃1024 | ˃1024 | 256 | 64 | 512 | 64 | 8 | 1024 | 0.5 | 2 | XDR |
| A52 | <4 | <4 | <8 | <8 | <<16 | <32 | <8 | <4 | <1 | <2 | <4 | <4 | <4 | <4 | 0.25 | 0.25 | S |
| A53 | <4 | <4 | <8 | <8 | <16 | 32 | <8 | <4 | <1 | <2 | <4 | <4 | <4 | <4 | 0.25 | 1 | S |
| A54 | <4 | <4 | <8 | 16 | <16 | <32 | <8 | <4 | <1 | <2 | <4 | <4 | <4 | <4 | 0.25 | 1 | S |
| A55 | 16 | 16 | >1024 | 512 | 64 | ˃1024 | ˃1024 | ˃1024 | 256 | 16 | 512 | 64 | 8 | 1024 | 0.5 | 4 | XDR |
| A56 | 16 | 16 | >1024 | 512 | 64 | ˃1024 | 1024 | ˃1024 | 256 | 16 | 512 | 32 | 8 | 1024 | 0.25 | 2 | XDR |
| A57 | 16 | 64 | >1024 | 128 | 256 | ˃1024 | 256 | ˃1024 | 128 | 32 | 512 | 64 | 8 | 256 | 0.5 | 4 | XDR |
| A58 | 8 | 32 | >1024 | 128 | 128 | >1024 | ˃1024 | ˃1024 | 256 | 32 | 1024 | 64 | 8 | 256 | 0.25 | 2 | XDR |
| A59 | 8 | 64 | >1024 | 64 | 128 | ˃1024 | ˃1024 | 256 | 128 | 8 | 512 | 64 | 16 | 1024 | 0.5 | 2 | XDR |
| A60 | 8 | 32 | >1024 | 128 | 128 | ˃1024 | ˃1024 | ˃1024 | ˃256 | 32 | 512 | 64 | 8 | 1024 | 0.25 | 4 | XDR |
| A61 | 32 | 16 | >1024 | 128 | 256 | >1024 | ˃1024 | ˃1024 | 64 | 32 | 512 | 64 | 8 | 256 | 1 | 4 | XDR |
| A62 | 32 | 16 | >1024 | >128 | 32 | >1024 | ˃1024 | ˃1024 | 128 | 8 | 512 | 32 | <4 | 1024 | 0.125 | 4 | XDR |
| A63 | 32 | 32 | >1024 | 128 | 256 | ˃1024 | ˃1024 | ˃1024 | 64 | 16 | 512 | 64 | 16 | 512 | 1 | 4 | XDR |
| A64 | 32 | 64 | >1024 | 128 | 128 | >1024 | ˃1024 | ˃1024 | 256 | 8 | 1024 | 128 | 16 | 1024 | 0.5 | 2 | XDR |
| A65 | 32 | 32 | >1024 | 128 | 128 | >1024 | ˃1024 | ˃1024 | 128 | 8 | 512 | 64 | 16 | 512 | 1 | 4 | XDR |
| A66 | 8 | 32 | >1024 | >128 | 64 | >1024 | ˃1024 | ˃1024 | 128 | 16 | 512 | 128 | 16 | 1024 | 0.5 | 4 | XDR |
| A67 | 16 | 64 | 512 | 64 | 512 | >1024 | ˃1024 | ˃1024 | 16 | 16 | 512 | 64 | 16 | >1024 | 1 | 2 | XDR |
| A68 | 8 | 64 | >1024 | 64 | 64 | ˃2048 | ˃1024 | ˃1024 | 64 | 16 | 512 | 128 | 16 | 512 | 0.5 | 4 | XDR |
| A69 | 32 | 0.5 | >1024 | 128 | 64 | 256 | ˃1024 | 256 | 256 | 32 | 1024 | 128 | 16 | 1024 | 0.25 | 0.25 | MDR |
| A70 | 64 | 128 | >1024 | 64 | 256 | ˃2048 | ˃1024 | ˃1024 | 128 | 32 | 512 | 64 | 8 | 256 | 0.5 | 2 | XDR |

*S, susceptible; MDR, multidrug-resistant; XDR, extensively drug-resistant.

**TABLE S3** Antimicrobial susceptibility of 70 clinical isolates of *A. baumannii*

| Antimicrobial  class | Antimicrobial  drug | MIC (µg/mL) |  |  | Isolate number (total n =70; %) for | | |
| --- | --- | --- | --- | --- | --- | --- | --- |
|  |  | MIC_range_ | MIC_50_ | MIC_90_ | Susceptible | Intermediate | Resistant |
| β-Lactams | Meropenem | <4-128 | 32 | 64 | 1 (1) | 11 (16) | 58 (83) |
|  | Imipenem | <4-128 | 32 | 64 | 0 (0) | 12 (17) | 58 (83) |
|  | Ceftazidime | <8->1024 | 128 | 256 | 0 (0) | 10 (14) | 60 (86) |
|  | Ceftizoxime | <8->1024 | 128 | 256 | 1 (1) | 9 (13) | 60 (86) |
|  | Cefoperazone-Sulbactam (2:1) | <16-512 | 128 | 512 | 3 (4) | 10 (14) | 57 (82) |
| Aminoglycosides | Amikacin | <32->1024 | >1024 | >1024 | 0 (0) | 11 (16) | 59 (84) |
|  | Gentamicin | <8->1024 | >1024 | >1024 | 0 (0) | 11 (16) | 59 (84) |
| Fluoroquinolones | Ciprofloxacin | <1->256 | 62 | 256 | 0 (0) | 11 (16) | 59 (84) |
|  | Levofloxacin | <2-64 | 16 | 32 | 3 (4) | 9 (13) | 58 (83) |
| Tetracyclines | Tetracycline | <4->1024 | 512 | 1024 | 1 (1) | 10 (14) | 59 (84) |
|  | Doxycycline | <4->256 | 64 | 128 | 0 (0) | 10 (14) | 60 (86) |
|  | Minocycline | 2-32 | 16 | 16 | 30 (43) | 16 (23) | 24 (34) |
| Tetracyclines (Glycylcyclines) | Tigecycline | 0.25-2 | 0.5 | 1 | 1 (1) | 69 (99) | 0 (0) |
| Macrolides | Azithromycin | <4->1024 | 512 | 1024 | 3 (4) | 9 (13) | 58 (83) |
| Polymyxins | Polymyxin B | <0.12-4 | 2 | 4 | 0 (0) | 37 (53 | 33 (47) |

**TABLE S4** Biofilm formation ability of 70 clinical isolates of *A. baumannii*

| Biofilm formation ability | Isolate number  (Total n=70) | Rate |
| --- | --- | --- |
| No | 6 | 8.57% |
| Weak | 42 | 60.00% |
| Medium | 10 | 14.29% |
| Strong | 12 | 17.14% |

**TABLE S5** MICs of 46 indole derivatives against six XDRAB isolates

| Indole derivative | MIC (μg/mL) | Indole derivative | MIC (μg/mL) |
| --- | --- | --- | --- |
| 5-Iodoindole | 64 | 3-Methylindole | 64 |
| 5-Fluoroindole | 64 | 4-Chloroindole | 64 |
| 6-Bromoindole | 64 | 5-Bromoindole | 64 |
| 6-Iodoindole | 64-128 | 4-Iodoindole | 64-128 |
| 5-Nitroindole | 128 | 7-Nitroindole | 128 |
| 5,6-Dihydroxyindole | 128 | 4-Fluoroindole | 128 |
| 7-Chloroindole | 128 | 6-Chloroindole | 128 |
| 4-Bromoindole | 128 | 5-Fluoroindole | 128 |
| 7-Fluoroindole | 128 | 7-Iodoindole | 128 |
| 5-Methylindole | 128 | 3,3'-Methylenediindole | ＞128 |
| 6-Methylindole | 128~256 | 7-Methylindole | 128~256 |
| 3-Methylindoline | 128~256 | 5-Trifluoromethoxyindole-2,3-dione | 256 |
| Indigo red | 256 | 3-Indolecarboxaldehyde | 256~512 |
| 4-Methylindole | 256 | 2-Methylindole | 256 |
| 2-Methylindoline | 256 | 5-Iododihydroindole-2,3-dione | 512 |
| 7-Hydroxyindole | 512 | 5-Hydroxyindole | 512 |
| 4-Hydroxyindole | 512 | 6-Hydroxyindole | 512 |
| Indole | 512 | 1-Methylindole | 1024 |
| Melatonin | 1024 | 3-Indoleacetic Acid | ＞1024 |
| Indole-3-acetate methyl ester | ＞1024 | Indigo Blue | ＞1024 |
| 3-Indoleacetonitrile | ＞1024 | 3-Indolepropionic acid | ＞1024 |
| DL-5-Hydroxytryptophan | ＞1024 | Midostaurin | ＞1024 |
| 1H-Indole-3-sulfonate potassium salt | ＞1024 | Vincamine | ＞1024 |

**TABLE S6** Primer sequences used for gene expression

| Gene |  | Primer (5'→3′) |
| --- | --- | --- |
| *16S rRNA* | Forward | CAG​CTC​GTG​TCG​TGA​GAT​GT |
|  | Reverse | CGT​AAG​GGC​CAT​GAT​GAC​TT |
| *abaI* | Forward | GAC​TGC​TAG​AGG​AAG​GCG​GAT​TTG |
|  | Reverse | AGA​CTA​CTA​CCC​ACC​ACA​CAA​CCC |
| *abaR* | Forward | TAA​ATG​TCG​GTT​GGG​CTC​AGT​CAA​G |
|  | Reverse | GCT​GGA​ATG​CAC​TGT​TTG​AGT​CAA​C |
